# Osteoarthritis of the knee or hip significantly impairs driving ability (cross-sectional survey)

**DOI:** 10.1186/1471-2474-15-20

**Published:** 2014-01-17

**Authors:** Ulf Krister Hofmann, Maurice Jordan, Ina Rondak, Petra Wolf, Torsten Kluba, Ingmar Ipach

**Affiliations:** 1 Department of Orthopaedic Surgery, University Hospital of Tübingen, Hoppe-Seyler-Strasse 3, Tübingen D-72076, Germany; 2 Institute for Medical Statistics and Epidemiology, University Hospital of the Technical University of Munich, Bau 523, Ismaninger Strasse 22, München D-81675, Germany

**Keywords:** Automobile driving, Reaction time, Osteoarthritis of the knee, Osteoarthritis of the hip, Total brake response time, Driver reaction time

## Abstract

**Background:**

Advising patients about when they can drive after surgery is common practice after arthroplasty of the knee or hip. In the literature, the preoperative braking performance values of the patients are frequently taken as the “safe” landmark. We hypothesised that osteoarthritis (OA), the most frequent reason for arthroplasty, already compromises the ability to perform an emergency stop. We expected that both Reaction Time (RT) and Movement Time (MT) as components of the Total Brake Response Time (TBRT), would be prolonged in patients with OA of the knee or hip in comparison with healthy subjects. We also expected maximum pressure levels on the brake pedal to be reduced in such cases.

**Methods:**

A real car cabin was equipped with pressure sensors on the accelerator and brake pedals to measure RT, MT, TBRT and maximum Brake Force (BF) under realistic spatial constraints. Patients with OA of the knee (right n = 18, left n = 15) or hip (right n = 20, left n = 19) were compared with a healthy control group (n = 21).

**Results:**

All measured values for TBRT in the control group remained below 600 ms. OA of the right hip or knee significantly prolonged the braking performance (right hip: TBRT p = 0.025, right knee: TBRT p < 0.001), whereas OA of the left hip did not impair driving ability (TBRT p = 0.228). Intriguingly, OA of the left knee prolonged RT and MT to the same degree as OA on the contralateral side (RT p = 0.001, MT p < 0.001).

**Conclusions:**

This study demonstrates that depending on the localisation of OA, driving capability can be impaired; OA can significantly increase the total braking distance. To ensure safe traffic participation the safety margin for TBRT should be strictly set, under our experimental conditions, at around 600 ms. Moreover, therapeutic approaches to OA, such as physiotherapy, and patients receiving surgery of the left knee should take into account that left knee OA can also impair driving ability.

**Trial registration:**

Clinical trial registration number: Project number of the ethics committee of the University of Tübingen: 268/2009BO2; 267/2009BO2.

## Background

Modern lifestyle largely depends upon individual flexibility ensured by the possibility of using a motorcar. With an ageing society in Western countries, this poses the crucial question: up to what age or physical condition can driving a car be considered safe?

One key element of safe traffic participation is the ability to perform an emergency stop and halt the car within a short distance. The distance covered by a car before coming to a complete stop (total stopping distance) comprises two principal components: braking distance and reaction distance (see Table 1). The braking distance is determined mostly by the speed, but also includes technical features of the vehicle, as well as the surrounding environment. Technical developments over the past decades have been able to shorten the braking distance, so that today, up to a speed of approximately 60 km/h, the reaction distance, i.e. the distance covered by the car under standard conditions until the driver reacts and triggers the actual braking process, is longer than the braking distance of the vehicle itself [1].

**Table 1:** Components of Total Brake Response Time

**1. *Reaction time***	**2. *Movement time***	**3. *Device response time***
- Sensation	- Lift the foot off the accelerator pedal and transfer it to the brake (*Foot Transfer Time*)	- Time it takes the device to engage once activated
- Perception/recognition	- Depress the pedal (*BrakePedal Travelling Time*)	
- Situational awareness		
- Response selection		
- Programming		

Total Stopping Distance = Reaction Distance + Braking Distance.

Reaction Distance = Total Brake Response Time x Speed of the Vehicle.

Adapted from Green, 2000.

The time it takes the driver to react is called “total brake response time” (TBRT), which can be further subdivided into the following categories, as suggested by Green, 2000 [2] and Spalding et al., 1994 [3]:

1. Reaction time (RT)

2. Movement time (MT)

3. Device response time (see Table 1)

Although brake assist systems can, to a certain extent, compensate for insufficient maximum pressures in modern vehicles, the brake force (BF) applied to the brake pedal is still an important factor for effective braking. However, BF and TBRT (comprising RT and MT) must be considered as highly variable among individuals and more importantly, influenced by numerous pathologies.

From an orthopaedic point of view, the primary disease of interest potentially impairing these parameters is osteoarthritis (OA) of a lower extremity. OA is a degenerative joint disease affecting articular cartilage and secondarily ligamental and capsular structures as well as the subchondral bone. It leads to pain, as well as reduced flexibility and mobility of the joint. In terms of prevalence, OA affects more than 70% of adults between 55 and 78 years of age in the USA [4]. Although difficult to quantify due to an undefined cut-off threshold, the incidence of symptomatic knee osteoarthritis is estimated at 1% per year, with a radiographic incidence of 2% per year [5,6]. Its symptomatic prevalence has been estimated to be about 12-16% in adults aged 45+ years [7,8]. Symptomatic OA-prevalence of the hip is considered to be about 10% in adults ≥45 years of age [9].

Due to the demographic changes in the population, it is assumed that OA-prevalence will largely increase over the coming years [4]. This will not only be accompanied by further financial burdens on the health care system [10,11], but will also affect a range of social mobility issues. It is therefore of considerable interest to address the question as to what extent OA compromises the ability to drive safely.

A wide range of possible TBRT limits has been proposed by different road authorities, ranging from 1500 ms [12] to just 700 ms [13,14]. The suggested limits are largely based on the premise that events can be “expected” or “unexpected”. When the event is expected, i.e. the driver is anticipating the signal, this leads to a much faster reaction than having to act upon an unexpected event. Green [2] suggests that when fully aware of the time and location of the brake signal, drivers can detect a signal and move the right foot from the accelerator to the brake pedal in about 700 ms, whereas response to “unexpected”, but common signals such as brake lights is about 1250 ms, and TBRT for “surprise” events roughly 1500 ms.

Moreover, many other factors such as driver age, gender, level of fatigue, drug intake, the design of the driver’s cab, or the nature of the signal can further influence reaction and response times [15-19].

However, these factors can be neglected when longitudinally testing the same collective pre- and post-interventionally. The same applies when comparing a potentially pathologic collective with a healthy group, although in this case confounders such as driver age and gender should be experimentally controlled. In many studies focusing on TBRT analysis, the impact of orthopaedic procedures has been evaluated by taking the preprocedure values as the safe value, thereby focussing on reconvalescence after surgery. Few studies had previously compared OA subjects with healthy controls; however those that did found significant differences in TBRTs between these two collectives [20].

In this study, braking performance of patients with OA of the knee and hip was analysed and compared with a healthy collective to evaluate the extent to which these pathologies compromise safe driving. We assessed not only TBRT with its components RT and MT, but also the applied force on the brake pedal. Moreover, since it was important for us to investigate the actual time required to react under realistic spatial constraints of a car cabin, we used a Volkswagen automobile body.

We first established reference values for RT, MT (= TBRT) and BF under our defined experimental conditions. We hypothesised that OA already compromises RT and MT (i.e. TBRT) when affecting the right knee or hip. We also expected maximum pressure levels on the brake pedal to be reduced in such cases. Moreover, we hypothesised that OA of the left knee or hip would not significantly impair TBRT and BF.

## Methods

### Procedure

**Figure 1:**
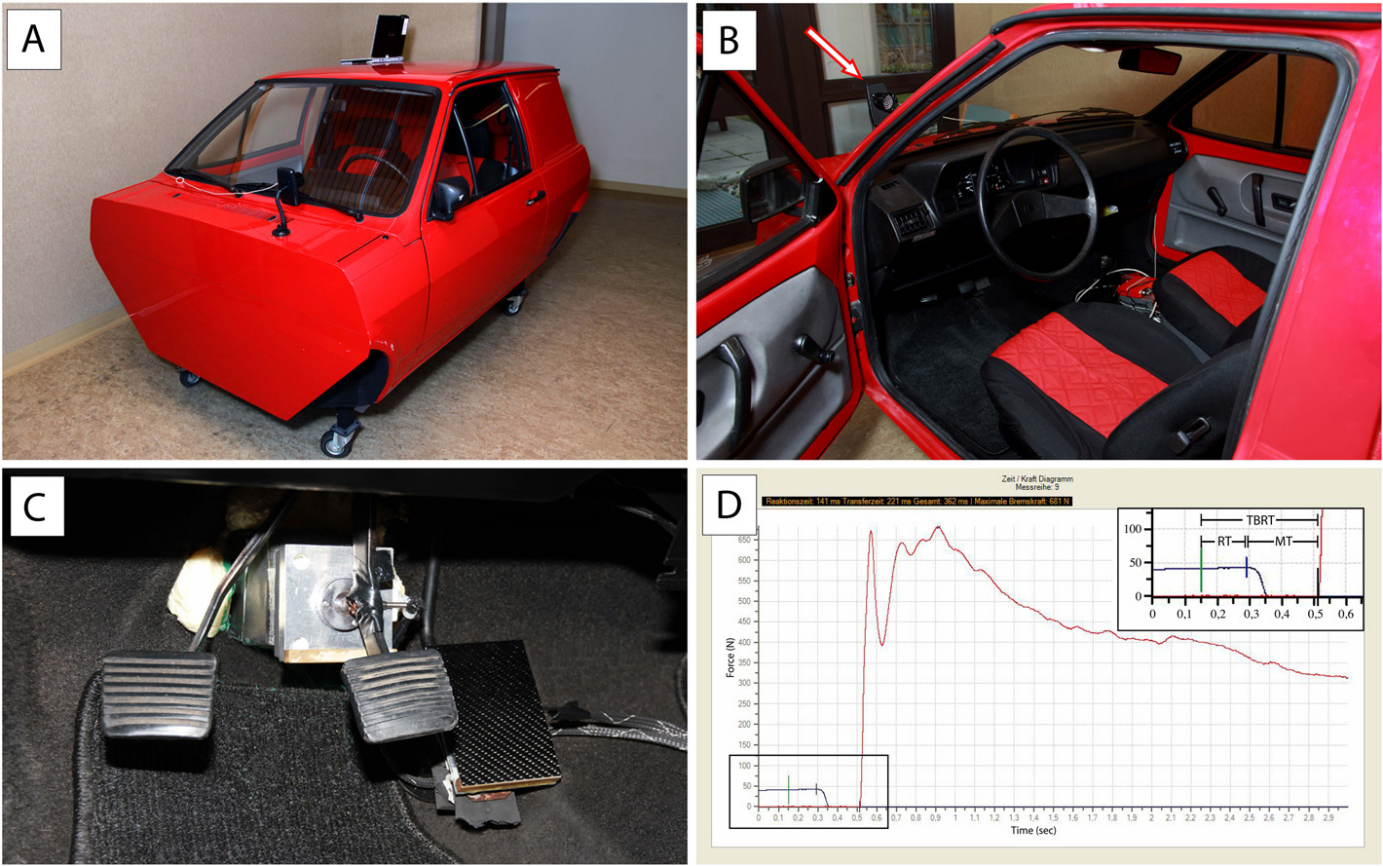
**Experimental setup and recorded data. (A)** Custom-made car simulator (VW Polo) for measuring total brake response time (TBRT), reaction time (RT), movement time (MT) and brake force (graph in D). **(B)** View of the driver cabin – the white arrow indicates the red flashlight. **(C)** Overview of the pedals with the accelerator pedal on the right and the brake pedal in the middle, both equipped with a pressure sensor, and the clutch pedal on the left. **(D)** Graph of computer output data, showing TBRT, RT and MT. The top right insert is an enlargement of the bottom left section of the graph. The green vertical line indicates the triggering of the red flashlight, the blue vertical line the beginning of pressure decrease (blue plot) on the accelerator pedal, and the black vertical line the beginning of pressure increase on the brake pedal (red plot).

We developed a brake simulator within a Volkswagen car cabin, thus creating real ergonomic conditions of a European middle-class car (Figure 1A, B).

Both the accelerator and brake pedal were equipped with a force transducer (KMB31, MEGATRON Elektronik GmbH & Co. KG) to register the applied force (Figure 1C). The transducer was directly clamped onto the accelerator pedal and set to a nominal load of 400 N. At the brake pedal, the sensor was attached behind the suspension of the pedal to increase measurement precision. This was necessary, since during rapid transfer of the foot from the accelerator to brake pedal, the latter is not always pushed centrally. Since higher pressures are reached on the brake pedal its nominal load was set to 2000 N. Signal output of the force transducers was connected to a measurement amplifier (EMA 3 DMS, MEGATRON Elektronik GmbH & Co. KG) and then sent to the MEphisto Scope (Meilhaus Electronic GmbH). This two-channel multifunctional module has an integrated voltmeter to register the signal coming from the force transducers.

The simulated emergency signal (see below) from a triggered red LED light that was placed in front of the windscreen at the driver’s eye level, was also received by the MEphisto Scope. These data were transmitted to a laptop equipped with a custom-made measuring programme developed according to the required specifications (Engineering office Michael Sawatzki). Calibration of the sensors was carried out according to standard procedures.

An electronic timer measured the elapsed time. The measured pressures and times were displayed on a computer screen visualised in the form of a diagram (Figure 1D).

The driver’s seat was always adjusted to each patient’s normal driving position with respect to seat inclination, head-rest and seat-pedal distance before testing [21]. Subjects were asked to wear footwear of their own choice that would correspond to footwear normally used when driving a car.

Subjects were asked to push the accelerator pedal continually, thus starting the computer-based registration. The supervisor then activated the red LED light at varying time intervals, using a hand control unit out of sight of the patient. Participants were instructed to consider this light as the simulated emergency situation and initiate full emergency braking. The right foot was thus lifted off the accelerator pedal, transferred to the brake pedal and maximum brake pressure applied, while the left foot rested on the clutch pedal. Absolute pressure levels were measured on the accelerator and brake pedal, allowing one to quantify the mode of lifting the foot off the pedal and depressing the brake pedal. The time elapsed between triggering the LED light and the start of reduced pressure on the accelerator pedal reflects the measured RT.

Various endpoints for MT have been described in the literature, ranging from the start of pressure increase (e.g. [20,22]), 100 N on the brake pedal [3,23], up to a minimum of 200 N being applied on the pedal [24,25]. We defined MT as from the start of reduced pressure on the accelerator until the start of increased pressure on the brake pedal.

**Figure 2:**
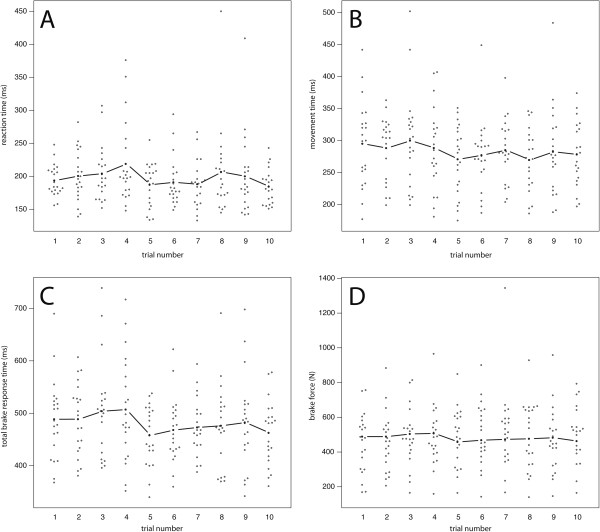
**Reference values for RT, MT, TBRT and BF under defined experimental conditions. (A)** RT, **(B)** MT, **(C)** TBRT and **(D)** maximum BF of the control group for ten iterations of the experiment. No significant learning curve or deterioration due to exhaustion is observed. White dots represent individual values; the black lines show their median.

RT, MT, TBRT, and maximum BF were measured 10 times by this procedure after the participant was familiar with the setup (3 practice trials). All subjects were given the same standardised instructions. No significant learning curve or deterioration due to exhaustion was observed in a control group (n = 21) for any of these parameters during the 10 repetitions, allowing their mean value to be used (Figure 2). The parameters gained with this experimental design are to be considered under the condition “expected”. Subjects were awaiting the signal and therefore no decision-making process as to whether to steer or to brake was required. The displayed signal was clear, yet no immediate danger suggested a vital urgency. The patient was not distracted by a cognitive load since there were no obstacles to consider.

### Statistical analysis

Statistical analysis was conducted using IBM SPSS Version 20 and R 2.13.2 (R Foundation for Statistical Computing). Distributions of variables within the study groups (i.e. control, right knee OA, left knee OA, right hip OA, left hip OA) were assessed by histograms, where deviations from the normal distribution (i.e. no symmetric distribution around the mean) were observed. Therefore a non-parametric approach was chosen. Continuous variables are presented as medians and ranges, and categorical variables as frequencies.

Demographic characteristics (i.e. age, body height, body weight, and body mass index (BMI)) and measurements of breaking performance (i.e. TBRT, RT, MT, and BF) were compared between all five study groups with the Kruskall-Wallis test or chi-squared test for gender. Post-hoc comparisons of the average braking performances of patients with OA of the knee or hip with the control group were carried out using exact Mann-Whitney-U tests.

All reported p-values are two-sided, with a significance level of 0.05, and have not been adjusted for multiple testing.

### Participants

Patients attending our department were asked at random to participate in the study. A norm collective was formed of 21 patients without clinical signs of OA of the lower extremity. 33 patients with OA of the knee (15 left, 18 right) and 39 patients with OA of the hip (20 left, 19 right) were measured. Inclusion criteria were clinically manifest OA of either the knee or the hip, flexibility of the hip of at least 90° flexion, a valid driving license and regular driving activity (at least 1x/week). Exclusion criteria were a neoplastic or infectious aetiology, coexisting neurologic disorders, acute trauma, a motor deficit of less than 4/5 on the British Medical Research Council scale for muscle power, arthrodesis of the ankle, and drug intake known to affect reaction time. Full departmental, institutional and ethical committee approval of the University of Tübingen/Germany were obtained before commencement of the study. Written informed consent was received from all subjects before participation.

## Results

A significant difference in body height (p = 0.041) and consequently also in BMI (p = 0.013) was found between all five groups, whereas no significant differences were observed in age (p = 0.368) and body weight (p = 0.260). No significant association was observed with gender and group (p = 0.849) (Table 2).

**Table 2:** Demographic Data

**Variable**	**Overall statistical significance**	**Control group**	**Study group**			
		**(n = 21)**	**OA left knee**	**OA right knee**	**OA left hip**	**OA right hip**
			**(n = 15)**	**(n = 18)**	**(n = 20)**	**(n = 19)**
Age* [yr]	p = 0.368 n.s.	68 (50-84)	74 (55-86)	68 (36-76)	68 (43-78)	69 (49-79)
Female [n]	p = 0.894 n.s.	10	9	11	12	10
Male [n]		11	6	7	8	9
Body weight* [kg]	p = 0.260 n.s.	82 (56-98)	82 (65-103)	91 (50-165)	86 (67-113)	79 (51-100)
Body height* [m]	**p = 0.041**	1.75 (1.58-1.90)	1.67 (1.50-1.76)	1.69 (1.60-1.88)	1.73 (1.60-1.88)	1.68 (1.53-1.92)
BMI [kg/m2]	**p = 0.013**	26.4 (21-29)	29.4 (25-39)	30.3 (18-50)	26.7 (24-39)	27.8 (20-33)

*The values are given as the median (min - max). Demographic characteristics were compared with the use of Kruskal-Wallis test or chi-squared test (gender). A p-value in bold type denotes a significant difference. n.s. = not significant.

**Figure 3:**
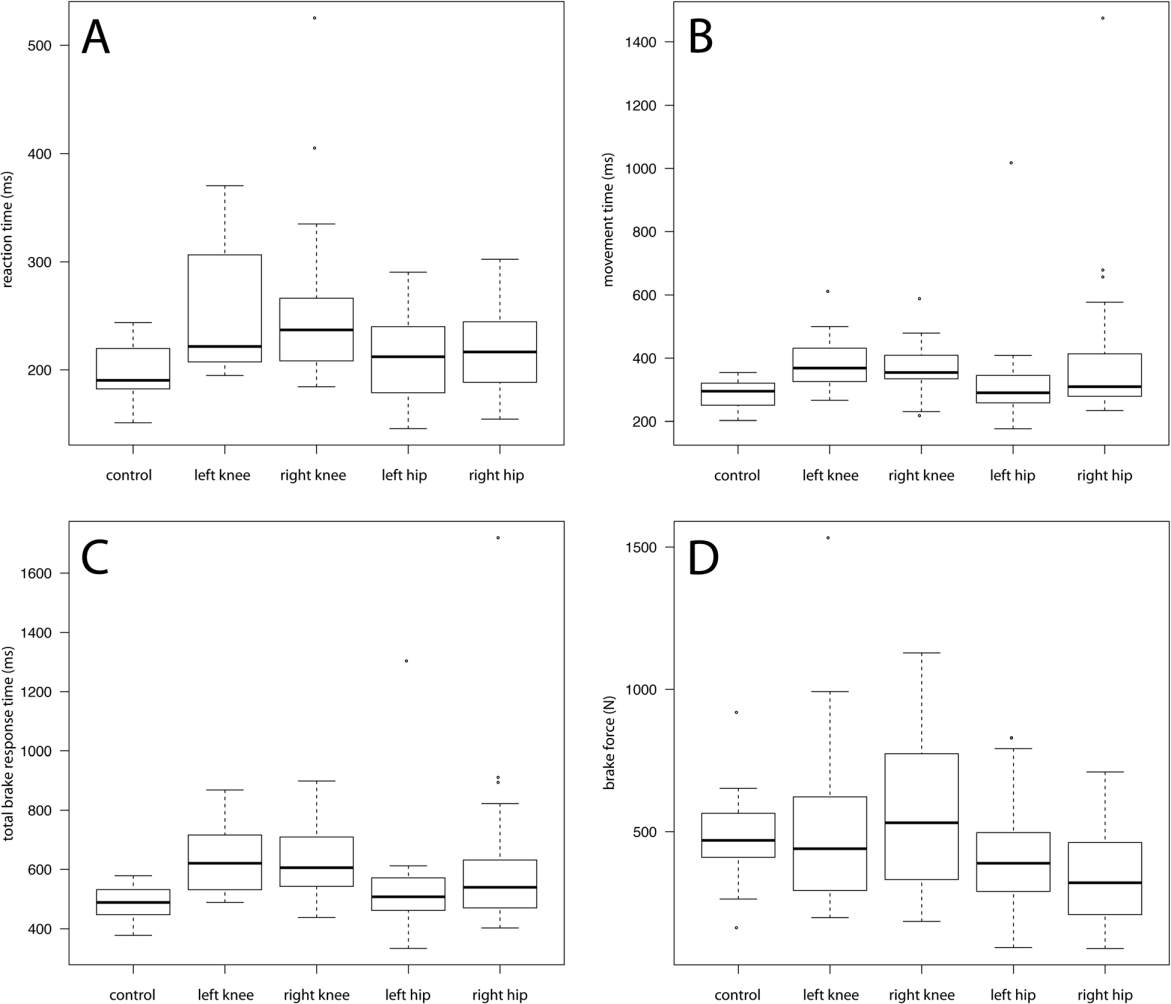
**Recorded results for all tested groups.** Box plots show **(A)** RT, **(B)** MT, **(C)** TBRT and **(D)** BF for the control group and the different OA patient groups. Data are expressed as median, interquartile range and extreme values.

The results of our testing are displayed in Figure 3, the statistical evaluation is shown in Table 3. The control group in our experimental setting recorded a median TBRT of 488 (378-578) ms. No subject in the control group was found to exceed a median TBRT of 600 ms and in only 14 out of the 210 individual tests performed by the 21 control subjects did single TBRT measurements slightly exceed 600 ms. Thus, under our experimental conditions, TBRT in the control group clearly remains below the general recommendations in the literature for maximum TBRT.

**Table 3:** Measured values for Total Brake Response Time, Reaction Time, Movement Time and Brake Force

**Variable**	**Control group (n = 21)**	**Left knee OA (n = 15)**	**Right knee OA (n = 18)**	**Left hip OA (n = 20)**	**Right hip OA (n = 19)**
TBRT* [ms] p-value	488 (378-578)	621 (488-868) **<0.001**	606 (437-899) **<0.001**	507 (334-1304) 0.228 n.s.	539 (403-1720) **0.025**
RT* [ms] p-value	190 (151-244)	222 (195-370) **0.001**	237 (184-525) **<0.001**	212 (146-291) **<0.001**	217 (154-302) 0.098 n.s.
MT* [ms] p-value	295 (203-354)	368 (267-611) **<0.001**	354 (218-588) **<0.001**	290 (177-1018) 0.527 n.s.	309 (234-1475) 0.078 n.s.
BF* [N] p-value	469 (163-919)	440 (197-1533) 0.680 n.s.	532 (184-1129) 0.477 n.s.	389 (93-830) 0.098 n.s.	320 (90-710) **0.022**

*The values are given as the median with the range in parentheses. P-values were calculated by means of the exact Mann-Whitney-U test using a two-sided 0.05 level of significance.

A p-value in bold type denotes a significant difference. n.s. = not significant.

*Abbreviations*: total brake response time (TBRT), reaction time (RT), movement time (MT), brake force (BF), osteoarthritis (OA).

The collective with OA of the right knee showed prolonged RT, MT and TBRT (each p <0.001) when compared with the control group; however, BF was not impaired. This is in contrast to subjects with OA of the right hip where BF was significantly reduced (p = 0.022). Median TBRT in this group was also found to be significantly prolonged (p = 0.025), although values were very heterogeneous. While most test subjects with OA of the right hip performed normally with TBRTs below 600 ms, 4 out of 19 subjects exceeded a TBRT of 800 ms.

Median TBRT of the subjects with OA of the left hip was statistically not different from the subjects in the healthy control group (p = 0.228), even if some patients were found to have a prolonged TBRT. In contrast to the group with right hip OA, BF was not altered significantly in these patients (p = 0.098).

For the group with OA of the left knee almost identical values to those on the contralateral side (right knee OA) were found for MT, RT and TBRT, with all of them significantly prolonged compared to the control group (p < 0001). As on the right side, BF was not reduced in the left knee OA collective.

## Discussion

In this study, we wanted to investigate whether preoperative OA of the knee or hip compromises safe traffic participation. The experimental setup comprised a European middle-class car cabin to imitate realistic conditions as closely as possible. The brake and accelerator pedals are arranged identically for both right- and left-hand traffic, and operated by the right foot.

Although to our knowledge no previous work had explicitly investigated the role of lower extremity OA on driving fitness, several publications have reported on the effect of OA on TBRT in the context of analysing patient collectives scheduled for joint operations. However, the findings were not unanimous. Spalding et al., 1994 [3] found no difference between patients with OA of the knee compared to subjects without OA, with measured TBRTs similar for OA on the left and right side.

In contrast, Hau et al. [25] described significantly increased preoperative TBRTs in a collective scheduled for right knee arthroscopy. Liebensteiner et al., [20] reported similar longer TBRTs for knee OA on both sides before total knee arthroplasty. In both groups values were significantly prolonged when compared with a norm collective [20], where the mean age was, however, younger by 13 years.

Our own study could confirm the latter findings. The most important result was that we observed equal impairment for both OA of the left (p < 0.001) and OA of the right knee (p < 0.001). The difference in median TBRT compared to the control collective was 133 ms on the left and 118 ms on the right side, resulting in a total braking distance increase of 3.7 m and 3.3 m respectively at 100 km/h. Hence both components of TBRT, that is RT and MT, were significantly longer in patients with knee OA, independently of the side affected. Nevertheless, median values for TBRT in patients with knee OA were clearly under the recommended margin of 700 ms.

Intriguingly, total brake force was not reduced in patients with knee OA (p = 0.680 n.s. left, p = 0.477 n.s. right). However, the fact that OA of the knee on both sides already significantly impairs TBRT might account for the fact that TBRT is apparently not crucially impaired after total knee arthroplasty when compared to the preoperative values [20,26].

In subjects with OA of the right hip, median TBRT was found to be significantly prolonged (p = 0.025), however only by about 51 ms, which represents increasing the braking distance by 1.4 m at 100 km/h. The total applied force on the brake pedal was found to be significantly reduced by 25% in patients with OA of the right hip (p = 0.022). The measured force of 320 N applied in this group is however, in our opinion, still sufficient to thoroughly trigger full braking. Moreover, statistical significance of the p-values for right hip OA braking performance has to be seen under the premise that no alpha adjustment was performed for multiple testing.

In contrast to left knee OA, OA in the left hip did not show any statistically different values for median TBRT or BF. These results are only partly in line with those published by MacDonald et al., [23], where significant differences were found in a preoperative collective for total hip arthroplasty on both left and right hip OA, the stronger effect being observed on the right side. However, in this study the tested sample size was rather small with only 9 subjects with OA on the left side, and the control collective was clearly younger than the pathologic group. The observed differences could possibly be due to the age difference between these two groups.

Although OA of the knee on both sides and OA of the right hip do compromise the ability to perform an emergency stop when compared to a healthy collective, based on current TBRT recommendations for an expected event, no recommendation against driving would appear necessary. The criteria for defining this capability to drive are, however, arbitrary. In the literature, the recommended maximum TBRT ranges from 1500 to 700 ms. Moreover, the conditions under which these times apply are rather vaguely defined. In addition to the state of expectation, many other factors including the cognitive load, environmental conditions, or the shape and threatening potential of the object are known to influence TBRT [2,15-19]. A recommendation for maximum TBRT can therefore only refer to a well-defined experimental setting. In our study not a single subject in the control group was found to exceed a median TBRT of 600 ms. We would therefore argue to set a maximum TBRT of around 600 ms instead of 700 ms when testing under our experimental conditions.

How can one interpret the fact that OA of the left knee also compromises driving ability? It has long been suggested that articular pain might produce an inhibitory effect on muscle reflexes [27]. For example, afferent stimuli from a damaged knee are associated with quadriceps inhibition [28], which can be observed ipsi- and contralaterally [29]. This phenomenon could possibly be explained by central processes influenced by pain sensation. Hurley et al. [28], however, suggested that arthrogenic muscle inhibition could be due to reduced efferent α-motoneuron output being modulated by afferent intrafusal muscle fibre signals. In addition, secondary spindle afferents projecting back to γ-motoneurons are supposed to innervate spindles in both homonymous and contralateral muscles (reviewed by Sjölander et al. [30]), thus changing the resting firing rate and stretch sensitivity of the afferents on both sides. Reduced muscle tone also on the contralateral side might thus lead to prolonged TBRT, even if OA is only manifest in the left knee.

To our knowledge, no information has been published so far on how to predict prolonged brake reaction times based on, for example, radiologic grading or clinical examination. In case of doubt we would therefore recommend individual testing in a drive simulator. However, it is doubtful whether good compliance can be expected in this respect, since surrendering a driving license means seriously compromising the quality of life. Testing is therefore probably rather of forensic interest.

### Study limitations

Although the gender ratio was similar in the different groups, the number of male and female subjects was not counterbalanced. However, we believe subject age to be the most important confounder in this experimental setting. We therefore strived to assess age-matched collectives. Median age of the subgroups was almost identical, with the exception of the left knee OA group, where the median was older by 6 years. The sample size was sufficient to find statistically significant and also clinically relevant differences between the groups. However, new allocation of a maximum TBRT should be retested in a larger collective before changing such a general recommendation.

Great efforts were made to create an experimental setting allowing for reliable emergency stop testing, but the complexity of everyday driving cannot be entirely reflected under laboratory conditions. Particularly the effect of a visual stimulus’ strength and urgency possibly overriding arthrogenic muscle inhibition would need to be investigated in further studies.

## Conclusions

In our study median TBRT for all four pathologic groups remained below the widely recommended limit of 700 ms. No recommendation against driving would therefore appear necessary in most cases of OA. Nevertheless, higher median values for TBRT in patients with OA of the knee, either right or left, extrapolate to a total braking distance increase of more than 3 m at 100 km/h compared to the control collective. This could represent the difference between a collision and the ability to stop in time.

In patients with OA of the right hip the median increase in braking distance was only about 1.4 m. However, four out of nineteen subjects exceeded a TBRT of 800 ms. This implies that any driving recommendation should be based on individual testing.

Notably, all subjects in the control group remained with their median TBRT clearly below 600 ms. We therefore believe that when testing under our moderate experimental conditions, the limit of TBRT for safe traffic participation should be reset from 700 ms down to a value below 600 ms.

Assuming such a parameter we would advise against driving in some cases of right hip OA, and in advanced stages of OA of the right or left knee. In particular, conservative therapeutic approaches to OA, such as physiotherapy, should take into consideration that left knee OA can also impair driving ability. Moreover, patients who have just received surgery of the left knee should be alerted to their potentially compromised ability to safely drive a car.

## Abbreviations

BF: Brake force; BMI: Body mass index; Fig.: Figure; km/h: Kilometres per hour; m: Metre; RT: Reaction time; ms: Millisecond; N: Newton; OA: Osteoarthritis; TBRT: Total brake response time; MT: Movement time.

## Competing interests

The authors declare that they have no competing interests.

## Authors’ contributions

UKH participated in the study design and drafted the manuscript; MJ was responsible for coordination and performance of the brake analyses and helped to draft the manuscript; PW participated in the design of the study and performed the statistical analysis; IR revised the statistical analysis; TK participated in the design and coordination of the study; II conceived the study, and participated in its design and coordination. All authors read and approved the final manuscript.

## Pre-publication history

The pre-publication history for this paper can be accessed here:

http://www.biomedcentral.com/1471-2474/15/20/prepub
